# Investigation of rs11568476 Polymorphism in the *SLC13A2* Gene in Turkish Patients with Hypocitraturia and Calcium-Containing Kidney Stones †

**DOI:** 10.3390/biomedicines13081985

**Published:** 2025-08-15

**Authors:** Ekrem Başaran, Dursun Baba, Yusuf Şenoğlu, Alpaslan Yüksel, Muhammet Ali Kayıkçı, Selma Düzenli, Ali Tekin

**Affiliations:** 1 Department of Urology, Faculty of Medicine, Duzce University, 81620 Duzce, Türkiye; drbaba28@hotmail.com (D.B.); aalii7@yahoo.com (M.A.K.); 2 Department of Urology, Faculty of Medicine, Marmara University, 34899 Istanbul, Türkiye; ysenoglu@yahoo.com; 3 Department of Urology, İstanbul Medeniyet University, 34720 Istanbul, Türkiye; dralpyuksel@gmail.com; 4 Department of Medical Genetics, Faculty of Medicine, Bolu Abant İzzet Baysal University, 14030 Bolu, Türkiye; duzenlis@gmail.com; 5 Department of Urology, Faculty of Medicine, Acıbadem University, 34752 Istanbul, Türkiye; aalitekin@hotmail.com

**Keywords:** calcium-containing kidney stones, hypocitraturia, SLC13A2 gene, rs11568476 polymorphism, NaDC1, genetic analysis

## Abstract

**Background and Objectives**: Hypocitraturia is a major risk factor for calcium-containing kidney stone disease. Citrate inhibits stone formation by binding calcium in the urine. The *SLC13A2* gene encodes the sodium-dependent dicarboxylate cotransporter 1 (NaDC1), a membrane transport protein that facilitates citrate reabsorption in the proximal renal tubules. Variants in this gene, such as rs11568476 (V477M), have been shown to significantly impair transporter activity. This study aimed to investigate the presence of the rs11568476 polymorphism in *SLC13A2* and its association with hypocitraturia in Turkish patients with calcium-containing kidney stones. To our knowledge, this is the first genetic study evaluating this polymorphism in a Turkish cohort. **Materials and Methods**: This prospective cross-sectional study included 90 patients diagnosed with calcium-containing kidney stones at Düzce University Faculty of Medicine, Department of Urology. Based on 24 h urinary citrate levels, patients were divided into two groups: normocitraturic (*n* = 38) and hypocitraturic (*n* = 52). Blood and 24 h urine samples were analyzed for biochemical parameters. The rs11568476 polymorphism in SLC13A2 was analyzed using Real-Time PCR. **Results**: There were no significant differences between the two groups in terms of age, gender, and most biochemical parameters. Serum uric acid levels were significantly higher in the hypocitraturic group (*p* = 0.002), whereas family history of stone disease was more prevalent in the normocitraturic group (*p* = 0.024). Genetic analysis revealed no polymorphism in the rs11568476 region; all patients exhibited the homozygous wild-type genotype (GG). **Conclusions**: No association was observed between the rs11568476 polymorphism and hypocitraturia in this cohort. The absence of the polymorphism suggests that this variant may be rare or absent in the Turkish population. These findings highlight the importance of investigating additional genetic and environmental contributors to hypocitraturia and nephrolithiasis through larger, multicenter studies.

## 1. Introduction

Citrate is a key inhibitor of calcium-containing kidney stone formation. It binds urinary calcium, forming soluble complexes and reducing supersaturation of calcium oxalate and calcium phosphate [1,2]. Hypocitraturia, defined as low urinary citrate excretion, is a common metabolic abnormality reported in 20–60% of patients with recurrent nephrolithiasis [3,4]. This condition may result from metabolic acidosis, gastrointestinal disorders, medications, or genetic predisposition [5,6].

The reabsorption of filtered citrate primarily occurs in the renal proximal tubule and is mediated by sodium-dependent dicarboxylate cotransporter 1 (NaDC1), a membrane transport protein encoded by the *SLC13A2* gene [7,8]. NaDC1 plays a central role in determining urinary citrate concentration, and its activity is regulated by systemic acid-base status. Functional alterations in NaDC1 can significantly affect urinary citrate levels and thereby influence stone risk [9,10].

Genetic variation within the *SLC13A2* gene has been proposed as a contributing factor to hypocitraturia. Several single-nucleotide polymorphisms (SNPs) have been identified, with varying effects on transporter function and expression [11]. Among them, the rs11568476 polymorphism, also known as the V477M variant, has been shown to reduce NaDC1 transport activity by approximately 90% in vitro, without affecting its expression [12]. Despite its functional relevance, the clinical implications of this SNP remain poorly understood, and few studies have examined its association with hypocitraturia or stone formation in human populations.

Most existing genetic studies have focused on alternative *SLC13A2* variants such as rs11567842 (I550V), which has been associated with hypocitraturia in East Asian cohorts [11,13]. However, there is a lack of data regarding the prevalence and significance of rs11568476 in other ethnic groups. Notably, its distribution in Turkish individuals remains unknown.

Türkiye is part of the so-called “stone belt,” with a high prevalence of urolithiasis. While several clinical and metabolic studies have been conducted in the country, genetic investigations into the etiopathogenesis of nephrolithiasis are limited. Understanding population-specific genetic susceptibility may provide insight into disease mechanisms and inform targeted prevention strategies [14,15].

The aim of this study is to determine the frequency of the rs11568476 polymorphism in the *SLC13A2* gene among Turkish patients with calcium-containing kidney stones and to assess its association with hypocitraturia. To our knowledge, this is the first study to evaluate this SNP in a Turkish cohort.

## 2. Materials and Methods

### 2.1. Study Design and Ethical Approval

This prospective cross-sectional study was conducted at the Department of Urology, Düzce University Faculty of Medicine, Türkiye, between March 2015 and June 2015. The study protocol was approved by the Clinical Research Ethics Committee of Düzce University (approval No. 2015/135, dated 3 March 2015), and written informed consent was obtained from all participants. This study complied with the ethical principles out-lined in the Declaration of Helsinki and institutional guidelines.

### 2.2. Patient Selection

**Figure 1:**
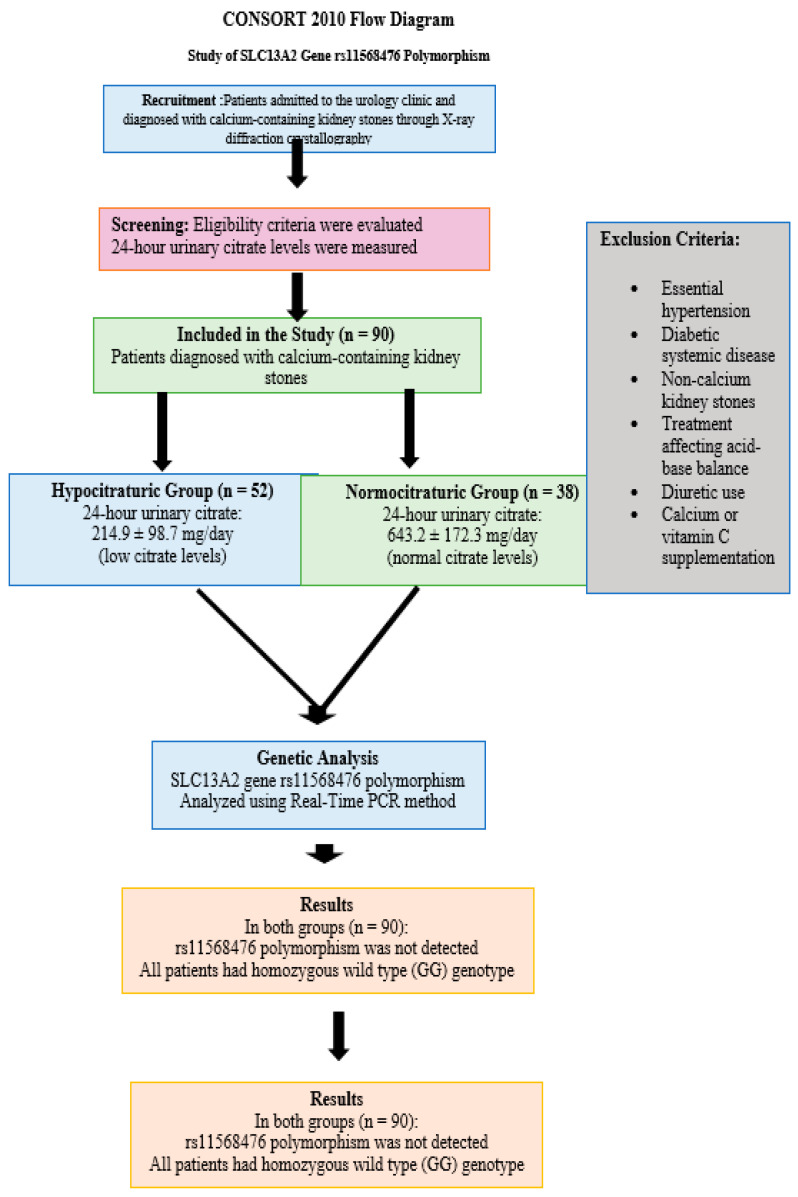
CONSORT 2010 flow diagram—study of *SLC13A2* gene rs11568476.

Patients presenting to the urology outpatient clinic with a diagnosis of calcium-containing kidney stones, confirmed via X-ray diffraction crystallography of stone samples, were evaluated for inclusion. Exclusion criteria were as follows: presence of any systemic disease other than well-controlled essential hypertension, history of non-calcium-based kidney stones, use of diuretics, calcium or vitamin C supplementation, and receipt of any pharmacologic treatment known to alter systemic acid-base balance (e.g., bicarbonate therapy or alkali treatment). Additionally, patients with secondary hypertension or those under antihypertensive therapies affecting renal citrate handling were excluded. Patient selection and enrollment are illustrated in the CONSORT 2010 flow diagram (Figure 1).

### 2.3. Data Collection and Dietary Control

Detailed clinical and demographic data were obtained through structured interviews and medical record reviews. Information collected included age, sex, height, weight, previous history of nephrolithiasis, history of surgical or medical treatments, systemic or metabolic disorders, and family history of kidney stones. Anthropometric measurements were performed using standardized clinical protocols. In order to minimize dietary variation that could influence urinary citrate levels, all patients were provided with written and verbal dietary instructions. These included restrictions on the intake of red meat, high-sodium foods, chocolate, spinach, leafy vegetables, tea, and coffee during the 48 h preceding the 24 h urine collection. Dietary adherence was not clinically supervised but was verified through direct patient interviews.

### 2.4. Biochemical Analysis

Venous blood samples were collected from all patients to measure serum creatinine, sodium, potassium, calcium, and uric acid concentrations. These analyses were performed using reagents from Biochemical Enterprise™, Milan, Italy (catalog no: CI8820), based on standardized enzymatic and colorimetric methods. Venous blood samples were collected from all patients to measure serum creatinine, sodium, potassium, calcium, and uric acid concentrations. These analyses were performed using an automated clinical chemistry analyzer (Architect c8000, Abbott Diagnostics™, Santa Clara, CA, USA), based on standardized enzymatic and colorimetric methods. Urine samples were collected over a 24 h period in acid-washed containers, and hydrochloric acid was added at the time of collection to reduce the pH below 3.0, ensuring citrate and oxalate stability. The following parameters were measured in the 24 h urine samples: citrate, oxalate, uric acid, calcium, and magnesium. All measurements were performed using photometric methods with commercial kits (Ben Biochemical Enterprise™, Milan, Italy; catalog no: CI8820). Based on 24 h urinary citrate excretion, patients were divided into two groups: normocitraturic (≥320 mg/1.73 m^2^/24 h) and hypocitraturic (<320 mg/1.73 m^2^/24 h).

### 2.5. Genetic Analysis

Genomic DNA was extracted from peripheral blood samples collected in Na-EDTA tubes using the High Pure PCR Template Preparation Kit (Roche Diagnostics™, Mannheim, Germany) following the manufacturer’s instructions. The rs11568476 polymorphism (V477M variant) of the *SLC13A2* gene was analyzed using a LightCycler 480 II Real-Time PCR system (Roche Diagnostics™) with LightSNIP-specific primers and HybProbe technology. Each 15 µL reaction mixture contained 10.4 µL PCR-grade water, 1 µL LightSNIP reagent mix, 2 µL FastStart DNA Master HybProbe (Roche Diagnostics GmbH, Mannheim, Germany), 1.6 µL 25 mM MgCl_2_, and 5 µL of DNA template. FastStart DNA Master HybProbe, 1.6 µL 25 mM MgCl_2_, and 5 µL of DNA template. Following centrifugation, PCR amplification and melting curve analysis were performed. Genotypes were classified based on melting temperature (Tm) peaks as wild-type homozygous (GG), heterozygous (AG), or homozygous mutant (AA).

### 2.6. Statistical Analysis

Statistical analyses were conducted using IBM SPSS Statistics version 22.0 (IBM Corp., Armonk, NY, USA). A priori power analysis indicated that at least 30 patients per group were required to detect statistically significant differences with 80% power and a significance level (α) of 0.05. Continuous variables were assessed for normal distribution using the Kolmogorov–Smirnov test and were expressed as mean ± standard deviation. Categorical variables were reported as frequencies and percentages. Group comparisons were performed using Student’s *t*-test for continuous variables and Pearson’s chi-square test for categorical variables. As all patients were found to carry the homozygous wild-type genotype (GG), Hardy–Weinberg equilibrium could not be assessed, and no genotype–phenotype association analysis could be performed.

## 3. Results

A total of 90 patients diagnosed with calcium-containing kidney stones were included and stratified into two groups based on their 24 h urinary citrate excretion: the hypocitraturic group (*n* = 52) and the normocitraturic group (*n* = 38). Table 1 summarizes the demographic and clinical characteristics of the study population.

There were no statistically significant differences between the two groups regarding age, gender distribution, height, or body weight (all *p* > 0.05). However, family history of urinary stone disease was significantly more prevalent in the normocitraturic group compared to the hypocitraturic group (60.5% vs. 36.5%, *p* = 0.024). This finding is somewhat counterintuitive and may suggest sample-specific or hereditary factors unrelated to urinary citrate levels.

Biochemical parameters revealed no significant differences in serum creatinine or calcium concentrations between the groups. Notably, serum uric acid levels were significantly elevated in the hypocitraturic group (5.08 ± 1.58 mg/dL vs. 4.12 ± 1.14 mg/dL, *p* = 0.002), suggesting a potential alternative metabolic contribution to stone formation in these patients. Urine specific gravity and pH values were comparable between groups (both *p* > 0.05).

Twenty-four-hour urine analysis results are presented in Table 2. As expected, urinary citrate excretion was markedly lower in the hypocitraturic group (155.3 ± 86.8 vs. 750.2 ± 238.1 mg/1.73 m^2^/24 h, *p* < 0.0001), confirming successful stratification. No significant differences were observed in 24 h urine volume, oxalate, uric acid, calcium, or magnesium excretion (all *p* > 0.05). Although urinary oxalate levels appeared higher in the normocitraturic group, the difference did not reach statistical significance (*p* = 0.097).

Genetic analysis of the rs11568476 region of the *SLC13A2* gene was successfully performed in all 90 patients using Real-Time PCR. Interestingly, none of the patients in either group carried the polymorphic variant. All participants (100%) were found to have the homozygous wild-type genotype (GG). As a result, no heterozygous (AG) or mutant homozygous (AA) individuals were identified. Due to the complete absence of allelic variation, genotype-based association analyses and Hardy–Weinberg equilibrium assessments could not be performed.

## 4. Discussion

This study investigated the presence of the rs11568476 (V477M) polymorphism in the *SLC13A2* gene in Turkish patients with calcium-containing nephrolithiasis, particularly focusing on its relationship with hypocitraturia, a known and common metabolic risk factor for kidney stone formation. Our main finding was the complete absence of the rs11568476 variant in all patients, regardless of urinary citrate status, suggesting this SNP may be rare or absent in the Turkish population [2,16].

Citrate is a central inhibitor of calcium stone formation, exerting its anti-lithogenic effect by complexing with urinary calcium, thus reducing ionized calcium availability and preventing crystal nucleation and aggregation [17]. The significance of hypocitraturia, defined as a reduction in urinary citrate excretion below reference values, has been well established as a recurrent and independent risk factor for calcium-based stone formation. Its etiology is multifactorial, involving dietary acid load, renal tubular defects, and genetic variants affecting citrate transporters, particularly *SLC13A2*, which encodes the sodium-dependent dicarboxylate cotransporter (NaDC1) in the proximal renal tubule [7,18].

The rs11568476 polymorphism has previously been associated with significantly reduced NaDC1 transport activity in functional studies, leading to the hypothesis that it may contribute to impaired urinary citrate excretion and, therefore, a predisposition to stone formation [19]. However, our study found no carriers of this polymorphism among the 90 patients analyzed, all of whom exhibited a homozygous GG (wild-type) genotype. While this may initially seem surprising, such absence likely reflects ethnic or population-specific differences in allele frequency. Prior reports of this variant have been limited and geographically restricted, with no established prevalence in Turkish or broader Middle Eastern cohorts [12].

This population-specific genomic profile is important for interpreting genetic risk and underscores the need for ethnically diverse genetic epidemiology studies. It is possible that *SLC13A2* polymorphisms other than rs11568476—such as rs11567842 (I550V)—may be more relevant in this context, as previously demonstrated in Thai and Japanese cohorts. These variants may exhibit distinct effects on transporter function and citrate handling, highlighting the polygenic and complex nature of hypocitraturia pathogenesis [20].

Another significant aspect of our findings was the lack of correlation between hypocitraturia and the studied polymorphism, supporting the view that this condition is not attributable to a single-gene defect in most cases [21]. Our study also revealed that serum uric acid levels were significantly higher in the hypocitraturic group, suggesting the presence of broader metabolic derangements. Hyperuricemia may reflect underlying acid-base disturbances or increased purine metabolism, both of which could influence citrate excretion through renal tubular pathways [22].

Additionally, a higher prevalence of family history of kidney stones in the normocitraturic group was a counterintuitive finding. While this may be due to sampling variation, reporting bias, or small subgroup sizes, it invites further exploration of non-citrate-related mechanisms of genetic inheritance in urolithiasis [23,24].

The absence of rs11568476 across the entire cohort raises questions regarding potential genotyping errors, as highlighted by one reviewer. However, our laboratory used validated protocols (Roche™ LightCycler 480, High Pure Template Kit, Roche Diagnostics GmbH, Mannheim, Germany), and internal controls confirmed procedural integrity. Moreover, melting curve analyses consistently showed wild-type signatures, making an analytical artifact unlikely. Nonetheless, replication in independent cohorts is warranted [25].

From a clinical standpoint, our findings suggest that rs11568476 is unlikely to serve as a useful genetic marker for hypocitraturia screening in Turkish stone formers. Given its rarity, routine genotyping for this variant appears to have limited utility in this population. Broader sequencing or SNP array-based approaches may be more informative, especially when combined with phenotyping for dietary habits, acid-base status, and renal function [26,27].

Therapeutically, the identification of individuals with hypocitraturia remains important regardless of genotype. Potassium citrate supplementation remains the cornerstone of treatment. However, novel approaches—including modulation of NaDC1 expression or function via SGLT2 inhibitors or lithium citrate—represent emerging strategies, and understanding genetic regulators of citrate transport could improve personalization of such interventions [28,29].

Despite its contributions, our study has several limitations. The relatively small sample size and lack of a healthy control group reduce statistical power and generalizability. The cross-sectional design prevents causal inference, and the evaluation of a single polymorphism limits the genetic scope. Furthermore, certain environmental and dietary variables that could impact citrate metabolism were not rigorously controlled or quantified. Methodologically, potential errors in 24 h urine collection and the lack of multiple measurements may have influenced citrate classification. Finally, the absence of rs11568476 may reflect a true population-specific rarity, but an undetected technical issue, although unlikely, cannot be completely ruled out.

## 5. Conclusions

This study investigated the presence and potential clinical relevance of the rs11568476 (V477M) polymorphism in the *SLC13A2* gene, which encodes the sodium-dependent dicarboxylate cotransporter 1 (NaDC1)—a key membrane transporter involved in renal citrate handling. Contrary to our initial hypothesis, none of the 90 Turkish individuals with calcium-containing kidney stones exhibited this polymorphism, regardless of their urinary citrate status.

This negative finding is important, as it highlights potential ethnic or population-specific differences in the genetic architecture of hypocitraturia. The absence of rs11568476 in our cohort suggests that this variant is likely rare or absent in the Turkish population and does not contribute significantly to the pathogenesis of hypocitraturia in this context. These results are aligned with the limited data available in the literature and further emphasize the need for population-specific genetic screening strategies in nephrolithiasis.

In addition, the significantly higher serum uric acid levels in the hypocitraturic group, along with the unexpected distribution of family history, indicate that multifactorial and potentially non-genetic mechanisms may underlie hypocitraturia and stone formation in these patients. These findings may warrant broader metabolic and genetic evaluation beyond single SNP analysis.

From a methodological standpoint, this study is strengthened by rigorous biochemical characterization and validated genotyping, yet limitations such as the modest sample size and lack of broader SNP screening should be acknowledged. Future research should consider expanding the genetic scope to include other functional variants of *SLC13A2*, such as rs11567842, and exploring additional genes involved in citrate metabolism and renal tubular transport.

In conclusion, while rs11568476 may have functional relevance in other populations, its absence in our cohort suggests limited utility as a biomarker for hypocitraturia or calcium stone disease in Turkish patients. Larger-scale, multiethnic studies integrating genomic, metabolomic, and clinical data will be crucial to fully elucidate the genetic landscape of hypocitraturia and guide personalized prevention and treatment strategies in urolithiasis.

## Figures and Tables

**Table 1:** Demographic and clinical characteristics of study groups.

Parameter	Hypocitraturic Group (*n* = 52)	Normocitraturic Group (*n* = 38)	*p*-Value
Age (years)	45.1 ± 13.2	44.2 ± 13.5	0.732
Gender (M/F)	31/21	20/18	0.509
Height (cm)	168.8 ± 6.6	169.0 ± 6.9	0.894
Body weight (kg)	76.3 ± 10.1	75.5 ± 9.5	0.705
Serum creatinine (mg/dL)	0.91 ± 0.27	0.86 ± 0.17	0.320
Serum calcium (mg/dL)	9.34 ± 1.41	10.22 ± 3.98	0.143
**Serum uric acid (mg/dL)**	**5.08 ± 1.58**	**4.12 ± 1.14**	**0.002**
Urine specific gravity	1016.1 ± 5.9	1017.0 ± 6.2	0.520
Urine pH	5.65 ± 0.90	5.49 ± 0.79	0.363
**Family history [*n* (%)]**	**19 (36.5%)**	**23 (60.5%)**	**0.024**

**Table 2:** 24 h urine analysis results.

Parameter	Hypocitraturic Group (*n* = 52)	Normocitraturic Group (*n* = 38)	*p*-Value
Urine volume (mL)	2112.3 ± 934.0	2308.4 ± 753.1	0.290
**Citrate (mg/1.73 m^2^/24 h)**	**155.3 ± 86.8**	**750.2 ± 238.1**	**<0.0001**
Oxalate (mg/1.73 m^2^/24 h)	33.0 ± 18.0	39.7 ± 19.6	0.097
Uric acid (mg/dL)	24.5 ± 12.6	23.1 ± 9.7	0.574
Calcium (mg/dL)	10.2 ± 6.1	9.6 ± 5.1	0.652
Magnesium (mg/dL)	5.04 ± 2.28	4.76 ± 2.30	0.576

## Data Availability

The raw data supporting the conclusions of this article will be made available by the authors upon request.

## Abbreviations

AE1	Anion Exchanger 1 (encoded by SLC4A1 gene)
ALDOB	Aldolase B, Fructose-Bisphosphate (gene)
ATP6V1B1	ATPase H⁺ Transporting V1 Subunit B1 (gene)
CA2	Carbonic Anhydrase II (gene)
CI	Confidence Interval
COS-7	Cell line derived from African green monkey kidney tissue
LiC	Lithium Carbonate
LiCit	Lithium Citrate
mg/dl	Milligrams per Deciliter
mg/1.73 m^2^/24 h	Milligrams per 1.73 square meters per 24 h
NaDC1	Sodium-dependent Dicarboxylate Cotransporter 1
NBCe1-A	Electrogenic Sodium Bicarbonate Cotransporter 1-A (SLC4A4)
OR	Odds Ratio
PCT	Proximal Convoluted Tubule
PCR	Polymerase Chain Reaction
PST-MR	Proximal Straight Tubule-Medullary Ray
SLC13A2	Solute Carrier Family 13 Member 2 (gene)
SNP	Single Nucleotide Polymorphism
SPSS	Statistical Package for the Social Sciences
Tm	Melting Temperature
